# Human umbilical cord mesenchymal stromal cells attenuate pulmonary fibrosis via regulatory T cell through interaction with macrophage

**DOI:** 10.1186/s13287-021-02469-5

**Published:** 2021-07-13

**Authors:** Zan Tang, Junxiao Gao, Jie Wu, Guifang Zeng, Yan Liao, Zhenkun Song, Xiao Liang, Junyuan Hu, Yong Hu, Muyun Liu, Nan Li

**Affiliations:** 1 Shenzhen Institutes of Advanced Technology, Chinese Academy of Sciences, Shenzhen, 518055 China; 2 Shenzhen Beike Biotechnology Co., Ltd., Shenzhen, Guangdong China; 3 National-Local Associated Engineering Laboratory for Personalized Cell Therapy, Shenzhen, Guangdong China

**Keywords:** Human umbilical cord mesenchymal stromal cell, Single cell RNA sequencing, Macrophage, Regulatory T cell

## Abstract

**Background:**

Pulmonary fibrosis (PF) is a growing clinical problem with limited therapeutic options. Human umbilical cord mesenchymal stromal cell (hucMSC) therapy is being investigated in clinical trials for the treatment of PF patients. However, little is known about the underlying molecular and cellular mechanisms of hucMSC therapy on PF. In this study, the molecular and cellular behavior of hucMSC was investigated in a bleomycin-induced mouse PF model.

**Methods:**

The effect of hucMSCs on mouse lung regeneration was determined by detecting Ki67 expression and EdU incorporation in alveolar type 2 (AT2) and lung fibroblast cells. hucMSCs were transfected to express the membrane localized GFP before transplant into the mouse lung. The cellular behavior of hucMSCs in mouse lung was tracked by GFP staining. Single cell RNA sequencing was performed to investigate the effects of hucMSCs on gene expression profiles of macrophages after bleomycin treatment.

**Results:**

hucMSCs could alleviate collagen accumulation in lung and decrease the mortality of mouse induced by bleomycin. hucMSC transplantation promoted AT2 cell proliferation and inhibited lung fibroblast cell proliferation. By using single cell RNA sequencing, a subcluster of interferon-sensitive macrophages (IFNSMs) were identified after hucMSC infusion. These IFNSMs elevate the secretion of CXCL9 and CXCL10 following hucMSC infusion and recruit more Treg cells to the injured lung.

**Conclusions:**

Our study establishes a link between hucMSCs, macrophage, Treg, and PF. It provides new insights into how hucMSCs interact with macrophage during the repair process of bleomycin-induced PF and play its immunoregulation function.

**Supplementary Information:**

The online version contains supplementary material available at 10.1186/s13287-021-02469-5.

## Introduction

Pulmonary fibrosis (PF) is a chronic progressive disease that happens when lung damaged and scarred due to an aberrant wound-healing process [26]. The thickened, stiff scar tissue makes blood oxygenation difficult and results in shortness of breath [8]. Numerous factors cause PF, including exposure to certain toxins, tobacco smoke, medical conditions, radiation therapy, viral infection, and some medications [4, 24, 33]. Mouse genetic studies suggest that lung epithelial cells, especially alveolar type 2 cells, play a key role in initiating the pathogenic process and excessive pulmonary fibroblast proliferation and extracellular matrix (ECM) deposition leads to the destruction of alveolar structure [3, 38, 41].

Pulmonary macrophages contain two populations: alveolar macrophages (AMs) which reside in the alveolar lumen and interstitial macrophages (IMs) that are located in the lung parenchymal tissue [19, 37]. Macrophages are crucial regulators of PF and undergo phenotypic and functional changes during the initiation, maintenance, and resolution phases following lung injury [1, 5, 39]. AMs are involved in ECM processing through the secretion of matrix metalloproteases [2, 7]. In the bleomycin-induced PF mouse model, IMs acquired a profibrotic phenotype with increased expression of CD206 (a mannose receptor) during the fibrotic phase [13, 27].

The role of mesenchymal stromal cells (MSCs) in the treatment of PF has been investigated in several studies [16, 20, 31]. MSC therapy has shown beneficial effects in animal experimental PF models by their potential immunosuppressive and anti-inflammatory activities [28, 30]. However, the detailed cellular and molecular mechanisms of MSCs in PF treatment remain to be elucidated.

In this work, we found that human umbilical cord mesenchymal stromal cells (hucMSCs) interact with macrophages and recruit regulatory T cells (Tregs) in damaged lungs. By using single cell RNA sequencing, we identified a subtype of interferon-sensitive macrophage with elevated *Cxcl10* expression levels. The increased CXCL10 in macrophages recruits Tregs to the lungs and in turn, suppresses the immune response in the lungs. Our study provides new insights into how hucMSCs interact with macrophage during the repair process of bleomycin-induced acute injury and play its immunoregulation function.

## Methods

### Mice

Eight- to about 12-week-old C57BL/6 male mice were purchased from the Guangdong Medical Laboratory Animal Center. All mice were maintained in an animal room with free access to food and water. All experiments were performed following the national guidelines for housing and care of laboratory animals and the protocol is reviewed and approved by the Animal Care and Use Committee of BeiKe Biotechnology, Ltd.

### Preparation of hucMSCs

Institutional review board approval from the ShenZhen Integrated Cell Bank was obtained for all procedures. Fresh umbilical cords were collected for scientific study from informed and consenting healthy donor. Mesenchymal tissue was scraped from Wharton’s jelly after blood vessels were removed. After cutting into pieces, the tissue was centrifuged at 600×*g* for 10 min at room temperature. The tissue was then washed with 0.9% saline solution and cultures at 37 °C with 5% CO_2_ in serum-free Dulbecco’s modified Eagle’s medium (DMEM). The primary hucMSCs were obtained after 10 days of culture. P4 hucMSCs were used in this study.

### Cell differentiation assay

For osteogenic, adipogenic, and chondrogenic differentiation, hucMSCs were cultured in relevant differentiation medium for 2~3 weeks by following the protocol of each medium and analyzed by staining with Alizarin Red, Oil Red O, and toluidine blue staining, respectively. The adipogenic differentiation medium (Cyagen, catalog#HUXUC-90031), osteogenic differentiation medium (Cyagen, catalog#HUXUC-90021), and chondrogenic differentiation medium (Cyagen, catalog#HUXUC-90041) were purchased from Cyagen in China.

### Bleomycin-induced mouse PF model

Eight- to about 12-week-old mice were anesthetized with isoflurane and received a single endotracheal dose of bleomycin sulfate (2 U/kg) at day 0 or 0.9% saline (45~60 μl) with equal volume. The bleomycin-treated mice were randomly divided into two groups. Half of the bleomycin-treated mice received a single i.v. (tail vein) dose of hucMSCs (5 × 10^5^ in 100 μl saline) at day 0. Half of the bleomycin-treated mice and saline-treated mice received equal volume of 0.9% saline via i.v. (tail vein) injection.

### Measurement of hydroxyproline levels

Lungs lobes were weighed, homogenized, and incubated in 6 M HCl at 110 °C for 2 to 6 h. The pH of hydrolyzed sample was adjusted to 6~8 by the NaOH. The hydroxyproline concentration of samples was determined by measuring the absorbance at 560 nm by a microplate reader (Molecular devices) and adjusted according to standard curves (Solarbio, catalog#BC0250).

### Tissue harvest and fixation

Mice were euthanized with i.p. injection of pentobarbital sodium. Mice were dissected to expose the diaphragm and the hearts were perfused with 0.9% saline through the right ventricle. The lungs were inflated to 25 cm H_2_O pressure with 4% paraformaldehyde (PFA) and were continually fixed in 4% PFA at 4 °C for 24 h. For H&E staining (see the following), lungs were embedded in paraffin. For immunolabeling (see the following), lungs were submerged in 30% sucrose for 24 h, and embedded in O.C.T. medium.

### Hematoxylin and eosin (H&E) staining

The H&E staining followed the basic protocol. Slides were dewaxed and rehydrated. Nuclei were stained by hematoxylin (Phygene, PH0516) for 4 min and the cytoplasm was stained by eosin (Phygene, PH0516) for 3 min. Slides were dehydrated in ascending alcohol solutions and cleared with xylene.

### Immunostaining

Fifteen-micrometer-thick sections were used for immunostaining. In brief, sections were blocked in 3% BSA/0.1% Triton X-100/PBS for 1 h at room temperature after O.C.T. was removed with PBS. Primary antibodies were diluted in blocks and incubated at 4 °C overnight. The sections were washed with 3%BSA/0.1% Tween-20/PBS 3 times; then, sections were incubated with secondary antibodies in blocks for 3 h at room temperature. All of the secondary antibodies were diluted in blocks at 1:400 dilutions.

### Antibodies

Chicken anti-GFP antibody (Abcam, ab13970, 1:500), Rabbit anti-Prospc (Millipore, ab3786, 1:500), Rat anti-Ki67 (ebioscience, 14-5698-82, 1:200), Rat anti-F4/80 (BioRad, MCA497GA, 1:200), APC anti-mouse CD326 (BioLegend, catalog#118214), PE/Cyanine7 anti-mouse CD45 (BioLegend, catalog#103114), PE/Cyanine7 anti-mouse CD31 (BioLegend, catalog#102418), PE anti-mouse F4/80 (BioLegend, catalog#123110), APC anti-mouse CD11c (BioLegend, catalog#117310), PerCP-Cyanine5.5 anti-human/mouse CD11b (Tonbo, catalog#65-0112), PE anti-mouse FOXP3 (BD biosciences, catalog#560408), APC anti-mouse CD4 (BD biosciences, catalog#553051), FITC Mouse IgG1 isotype control (BD biosciences, catalog#555748), PE Mouse IgG1 isotype control (BD biosciences, catalog#555749), APC Mouse IgG1 isotype control (BD biosciences, catalog#555751), FITC anti-human CD90 (BD biosciences, catalog#555595), PE anti-human CD73 (BD biosciences, catalog#550257), APC anti-human CD105 (BD biosciences, catalog#562408), FITC anti-human CD45 (BD biosciences, catalog#555482), PE anti-human CD34 (BD biosciences, 550761), Alexa Fluor 488 Donkey anti Chicken (Jackson Immuno Research, catalog#703-545-155), Alexa Fluor Cy3 Donkey anti Rat (Jackson Immuno Research, catalog#712-165-153), and Alexa Fluor 488 Donkey anti Rabbit (Jackson Immuno Research, catalog#711-545-152).

### Lung dissociation for flow cytometry

Lungs were perfused with saline to remove the blood cells, then inflated intratracheally with 1.5 ml enzyme solution containing neutral protease (Worthington-Biochem, catalog#LS02111, 5U/ml), Collagenase Type I (Gibco, catalog#17100-017, 200U/ml), elastase (Worthington, catalog#2294, 4U/ml), and DNase I (Roche, catalog#10104159001, 0.33U/ml) and soaked in the same solution for 45 min at room temperature. The digested lung tissues were gently torn into small pieces and shaken in Dulbecco’s modified Eagle medium (DMEM, Gibco) containing 10% FBS for 10 min at room temperature. After filtering through 100 μm and 40 μm strainers, centrifuged cells were incubated with 10 mL red blood cell lysis buffer to remove red blood cells. After centrifugation, cells were resuspended in DMEM for staining and FACS analysis (BD FACSAria™ II).

### EdU proliferation assay

For assessing cell proliferation in vivo, EdU (Thermo Fisher Scientific, Catalog#E10415) was administered to mice by intraperitoneal injection at 50 mg of EdU per kg of the mouse (weighed 24 h prior to sacrifice). EdU incorporation was detected using the Click-iT EdU Alexa Fluro 488 flow cytometry assay kit (Invitrogen, catalog#C10632).

### Immunostaining for flow cytometry

For AT2 EdU proliferation assays, cells were fixed and permeabilized by using fixation and permeabilization solutions (BD biosciences, catalog#51-2090KZ). For lung fibroblast proliferation assays, cells (10^6^~10^7^) were stained with APC anti-mouse CD326 antibody, PE/Cyanine7 anti-mouse CD45 antibody, and PE/Cyanine7 anti-mouse CD31 antibody. EdU staining was performed by using the Click-iT EdU Alexa Fluro 488 flow cytometry assay kit according to the manufacturer’s instructions. Briefly, cells were fixed by using Click-iT™ fixative buffer for 15 min after cell surface staining and then were permeabilized Click-iT™ saponin-based permeabilization and wash reagent for 15 min. The cell pellets were then incubated with Click-iT™ Plus reaction cocktail for 30 min before flow cytometry analysis.

### qRT-PCR

Total RNA was extracted from lungs using the RNeasy mini kit (QIAGEN, catalog#74104) according to the manufacturer’s instructions. cDNA was made using M-MLV reverse transcriptase (Invitrogen, catalog#28025-013). qRT-PCR was performed using SYBR Green Realtime PCR Master Mix (TOYOBO, catalog#QPK-201) in LightCycler 480 instrument.

**Table:** 

Gene	Forward	Reverse
*Irf1*	AGGCAAACTTCCGTTGTG	TTTCCTCTGGTTCCTGGTG
*Irf7*	GGGACCTCTTGCTTCAGGTT	AGGGTTCCTCGTAAACACGG
*Ifit1*	TCCGTAGGAAACATCGCGTA	TGTTGCTTGTAGCAGAGCCC
*Ifit2*	TCATCCAGCAACAGCATCC	CCAGATAAGCCTGAGCCTTTG
*Ifit3*	CAAGGTGAGACAAGTTTGCC	GCTCGTTCATTTCTTCCACAC
*Ly6c2*	CAGTGCTACGAGTGCTATGG	GACGGGTCTTTAGTTTCCTTC
*Isg15*	CGATTTCCTGGTGTCCGTGA	AGACCCAGACTGGAAAGGGT
*Rsad2*	ATTCTGGATGTTGGCGTGGA	ATACTTTCCGCCACGCTTCA
*Ifi205*	ATGCTACAGTGGCTACAGTGAG	ATCTCCAGGATGCCTTTG
*Ifi211*	TGTTCTGCTGAGAGGACTTG	TCTGAATCGTGGTGTATTGC
*Ifi204*	TCATCTAACATTCCTTCGGC	TGAGCACCATCACTGTCAG
*Ifi203*	GCCTCCAGAATCCTCATAAG	TCACTTGTTTGGGACCTTG
*Arg1*	TTGTGAAGAACCCACGGTC	TTCCAACTGCCAGACTGTG
*Mrc1*	TGACTTCATCTTCTCCCAGC	CCATCCTTGCCTTTCATAAC
*Tgm2*	CGACCTATGCCAAGAGAAAC	CCTCCTCCACATTGTCAGAC
*Nos2*	CAGCCCAACAATACAAGATGAC	GGGATTCTGGAACATTCTGTG
*Cxcl9*	TCTTGGGCATCATCTTCC	ATCTCCGTTCTTCAGTGTAGC
*Cxcl10*	TTTCTGCCTCATCCTGCTG	CGCAGGGATGATTTCAAG
*Chil3*	GACTTGCGTGACTATGAAGC	TGACGGTTCTGAGGAGTAGAG
*Gapdh*	TGCTGAGTATGTCGTGGAGTC	GGTTCACACCCATCACAAAC

### Live animal imaging

hucMSCs were labeled with the XenoLight DiR (1,1′-dioctadecyltetramethyl-indotricarbocyanine iodide; “DiR,” Perkin Elmer, Catalog#125964) for 25 min at 37 °C at a concentration of 15 μM in PBS. Following labeling, cells were washed twice in 0.9% saline solution to remove unbound dye before i.v. injection. Mice were imaged with a NightOWL in vivo imaging system.

### Macrophage and hucMSCs co-culture

hucMSCs (1 × 10^4^/cm^2^) were seeded in a 24-well plate 24 h before co-culture. Macrophages were sorted from the mice lung 1 day after bleomycin treatment and co-cultured with hucMSCs (10:1). The hucMSCs and macrophages were cultured in RPMI-1640, 10% FBS, and 1% Penicillin/Streptomycin. mRNA was extracted after co-culture for 72 h.

### Single cell RNA sequencing

Lung single cell suspensions were made according to the protocol described above (lung dissociation for flow cytometry) and then processed following the 10X genomics protocol. We used the Seurat R package (version 3.2.3) as the tool for downstream data analysis and processing. Cells less than 300 expression genes were removed and genes that were not expressed in any cells were excluded. To safeguard against undetected doublets, we kept cells with UMIs < 60000. Also, we removed the cells with high expression of mitochondrial and ribosomal genes (according to the proportion of mitochondria and ribosomes in the gene-barcode matrix, 25% and 1% for mitochondria and ribosomes, respectively). Considering the large range of UMI counts for each cell, we normalized the UMI counts. We used the log normalization method to normalize the UMI of cell i on gene j by the formula:

$$ \mathrm{n} orm\ {UMI}_{\left(i,j\right)}=\log \left(\frac{UMI_{\left(i,j\right)}}{\sum_i UMI}\times \mathrm{10,000}\right) $$

For dimensionality reduction and clustering analysis, we scaled our normalized UMI data by the formula:

$$ scale\ {UMI}_{\left(i,j\right)}=\frac{UMI_{(ij)}-\mu }{\sigma } $$

where μ and σ are the mean and standard deviation of UMIs in cell i, respectively. The two methods are integrated into the Seurat R package.

Before clustering analysis, we reduced the dimension of the gene-barcode matrix. To capture biological signals from the single cell data, we selected the top 2000 high variation genes according to the variance stabilizing transformation (VST) method and performed the principal component (PC) analysis among them. We selected the top 20 PCs by evaluating the standard deviation across PCs. We used the top 20 PCs for clustering analysis and visualization.

In cluster analysis, we constructed a shared nearest neighbor graph based on k-nearest neighbors calculated from the 20 PCs of the scaled data (k = 20) and used the Louvain algorithm as a modularity function optimizer to determine the number of clusters (resolution, 0.5).

### Differential expression analysis

The differentially expressed genes between one cell group and other cell groups were analyzed by running the Wilcoxon Rank-Sum test. We set the average log fold change threshold to 0.25 and limited the expression of genes > 10% in the two compared cell groups. Genes with FDR-adjusted *P* values < 0.05 were retained.

### Cell type annotation and macrophage screening

After determining the differentially expressed genes of each group of cells, we annotated the cell types based on the top 20 genes with log-fold changes in each group of cells (Table S1). We referred to the manually curated resource of cell markers database CellMarker, the cell type markers database PanglaoDB.

To ensure the accuracy of the selected macrophages, we conducted two rounds of clustering and differential gene analysis. Cells with high expression levels of T cell markers (*Trbc2*, *Cd3d*, and *Cd3e*) and B cell makers (*Igkc* and *Ighm*) were excluded. Overall, we have 829 macrophages in bleomycin group and 1016 macrophages in hucMSCs treated group.

### Statistics

All data are presented as mean ± SD (as indicated in figure legends). Experimental analyses were not blinded. Unless otherwise mentioned, most of the data presented in figure panels are based on at least three independent experiments. We used two-tailed Student’s *t*-tests to assess differences between means. *p*-values were depicted as follows: *p < 0.05, **p < 0.01, and ***p < 0.001.

## Results

### Human umbilical cord mesenchymal stromal cells can ameliorate bleomycin-induced PF

**Fig. 1:**
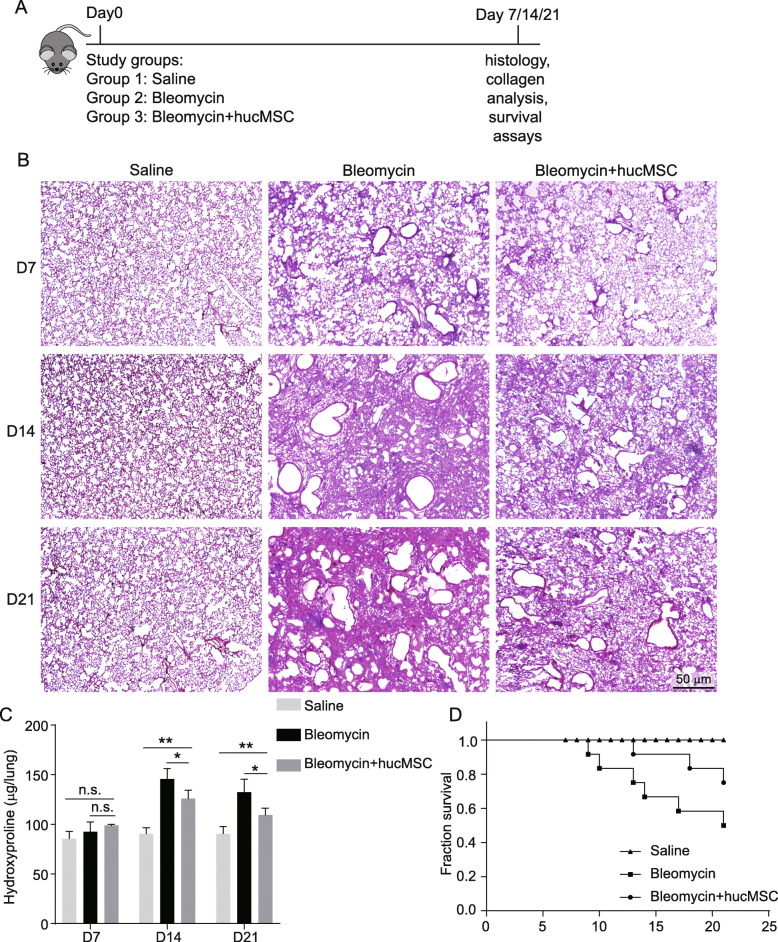
hucMSCs alleviated bleomycin-induced pulmonary fibrosis. **A** Scheme of the experimental timeline for the bleomycin-induced pulmonary fibrosis model and hucMSC infusion. **B** H&E-stained lung sections from saline-treated mice, bleomycin-treated mice, and hucMSC-treated mice at various time points. **C** Hydroxyproline contents of saline-treated mice lungs, bleomycin-treated mice lungs, and hucMSC-treated mice lungs at various time points (mean ± SD, *n* = 3 mice per group). **D** Survival curves of saline-treated mice, bleomycin-treated mice, and hucMSC-treated mice (*n* = 12 mice per group). *p < 0.05, **p < 0.01, n.s. no significant difference, Student’s *t*-test

To evaluate the role of human umbilical mesenchymal stromal cells (hucMSCs) on PF, mice were administered a single dose of bleomycin (2 U/kg) intratracheally on day 0 (Fig. 1A). Concurrent with bleomycin instillation, treatment groups were transplanted a single dose of hucMSCs via tail vein injection. hucMSCs were positive for MSC markers (CD90, CD73, and CD105) and were negative for hematopoietic markers (CD45 and CD34) (Figure S1A-1I). Meanwhile, hucMSCs could differentiate into adipocytes, chondrocytes, and osteocytes (Figure S1J-1L). Histologic evaluation of lung tissue from mice at 7, 14, and 21 days after bleomycin treatment revealed that hucMSCs can attenuate bleomycin-induced PF (Fig. 1B). Consistent with the lung histology results, collagen content was lower in hucMSC-transplanted mice lungs at 14 and 21 days compared to the bleomycin group (Fig. 1C). Furthermore, hucMSC-transplanted mice were more resistant to bleomycin-induced lung injury and exhibited a significant decrease in mortality relative to the bleomycin group (Fig. 1D). These results demonstrated that hucMSCs can ameliorate bleomycin-induced PF.

### hucMSCs promote lung regeneration after bleomycin treatment

**Fig. 2:**
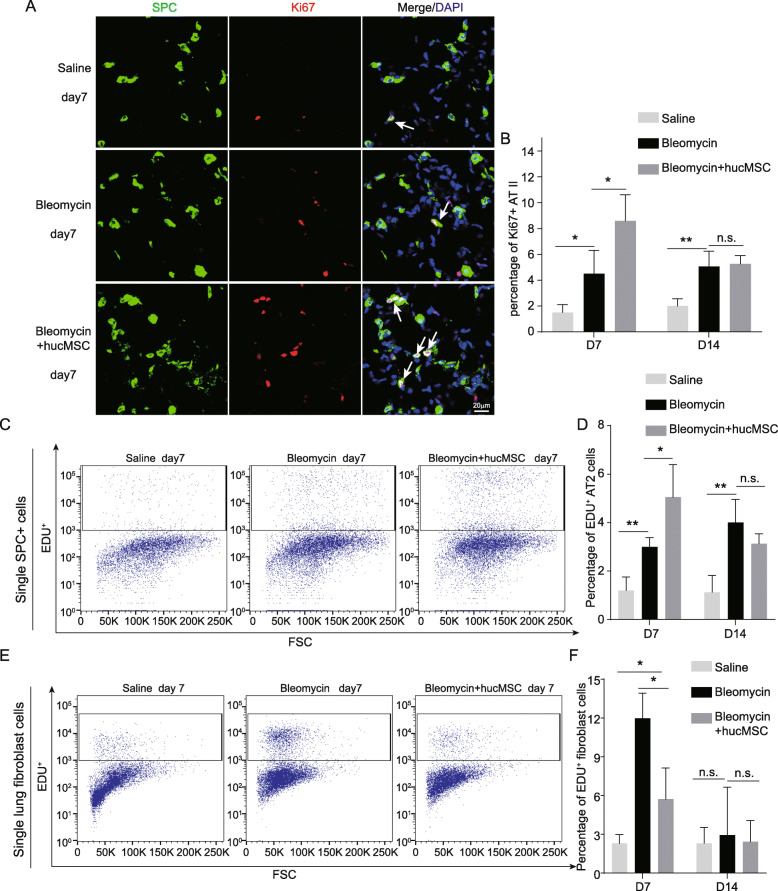
hucMSC infusion promoted lung regeneration. **A** Day 7 after bleomycin treatment, lung sections were stained with antibodies against proSPC and Ki67. White arrowheads indicate proliferating alveolar type 2 cells. **B** The percentage of Ki67-positive alveolar type 2 cells (mean ± SD, *n* = 3 mice per group). **C** Flow cytometry analysis of EdU incorporation in alveolar type 2 cells in saline-treated mice lungs, bleomycin-treated mice lungs, and hucMSC-treated mice lungs on day 7 and day 14. **D** The percentage of EdU-positive alveolar type 2 cells (mean ± SD, *n* = 3 mice per group). **E** Flow cytometry analysis of EdU incorporation in lung fibroblast cells in saline-treated mice lungs, bleomycin-treated mice lungs, and hucMSC-treated mice lungs on day 7 and day 14. **F** The percentage of EdU-positive lung fibroblast cells (mean ± SD, *n* = 3 mice per group). *p < 0.05, **p < 0.01, n.s. no significant difference, Student’s *t*-test

PF is associated with impaired alveolar type 2 (AT2) cell proliferation which can inhibit the repair of the damaged epithelium [23, 34, 38, 41]. To determine if hucMSCs administration affects AT2 proliferation in vivo, we stained saline-treated mice lung, hucMSCs-transplanted mice lung, and bleomycin-treated mice lung with the cell proliferation marker Ki67 and AT2 cell marker SPC. On day 7 after bleomycin treatment, we found that a greater number of SPC positive cells were stained with Ki67 in hucMSC-transplanted lungs than in lungs instilled with bleomycin (Fig. 2A, B). Similarly, flow cytometry revealed a significant increase in EdU incorporation in AT2 of hucMSC-transplanted lungs at day 7 compared to bleomycin-instilled lungs (Fig. 2C, D). The abnormal proliferation of lung fibroblasts contributes to the initiation and progression of PF [32]. However, lung fibroblasts are heterogeneous and consist of multiple subtypes such that there is no consensus on a lung fibroblast marker [40]. Therefore, we used EpCAM^-^CD31^-^CD45^-^ cells to represent lung fibroblasts in our subsequent studies by excluding lung epithelial cells (EpCAM+), endothelial cells (CD31+), and hematopoietic cells (CD45+), respectively. To assess the proliferation of fibroblasts in lungs, we quantified EdU incorporation in EpCAM^-^CD31^-^CD45^-^ cells on day 7 following bleomycin instillation (Figure S2A). After hucMSC transplantation, the lung fibroblast proliferation rate was lower on day 7 compared to the bleomycin-treated mice (Fig. 2E, F). However, the AT2 and fibroblast proliferation rates were not different between the hucMSCs-transplanted group and the bleomycin-treated mice at day 14 post bleomycin instillation (Fig. 2B, D, F). The AT2 and lung fibroblast proliferation data showed that hucMSCs promoted the alveolar stem cell repair process and inhibited abnormal lung fibroblast proliferation which contributed to the attenuation of bleomycin-induced PF.

### hucMSCs interact with macrophage in mouse lung after infusion

**Fig. 3:**
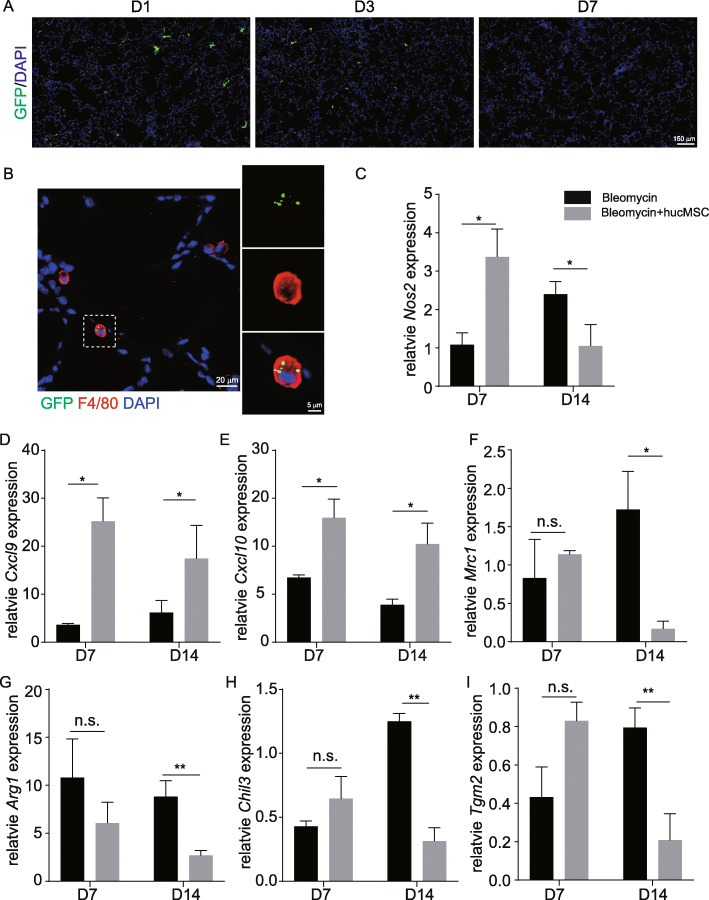
hucMSCs interacted with macrophages after infusion. **A** Immunofluorescent labeling of lung sections after GFP-labeled hucMSC infusion in bleomycin-treated lungs at various time points. **B** Lung sections were stained with antibodies against GFP and F4/80. **C**–**I** *Nos2*, *Cxcl9*, *Cxcl10*, *Mrc1*, *Arg1*, *Chil3*, and *Tgm2* expression levels in bleomycin-treated lungs and hucMSC-treated lungs on day 7 and day 14 by whole lung qRT-PCR. *Nos2*, *Cxcl9*, and *Cxcl10* expression levels (mean ± SD, *n* = 3 mice per group) were significantly increased in hucMSC-treated lungs compared to bleomycin-treated lungs at day 7 (**C**–**E**). *Mrc1*, *Arg1*, *Chil3*, and *Tgm2* expression levels (mean ± SD, *n* = 3 mice per group) were significantly decreased in hucMSC-treated lungs compared to bleomycin-treated lungs at day 14 (F-I). The expression level of markers and cytokines in bleomycin-treated lungs and hucMSC-treated lungs was compared to saline-treated mice lungs. *p < 0.05, **p < 0.01, n.s. no significant difference, Student’s *t*-test

To investigate how hucMSCs function in vivo, we tracked the behavior of hucMSCs by labeling them with a live cell dye, DiR. DiR-labeled hucMSCs aggregated in the lung soon after hucMSC injection without notable differences between mice treated with saline and bleomycin, and almost all hucMSCs were eliminated from the mice within 7 days in both groups (Figure S3A). To track the behavior of hucMSCs in greater detail, hucMSCs were transfected with membrane green fluorescent protein (mGFP) via lentivirus. Many mGFP-labeled cells were observed in lung sections on day 1, but were far less abundant at day 3 after injection, and were absent at day 7 which was consistent with the DiR results (Fig. 3A). It has been shown that mesenchymal stromal cells can modulate macrophage phenotypes and were phagocytosed by macrophages in other model systems. To determine if hucMSCs interacted with macrophages in vivo after infusion, we co-immunolabeled the lungs with antibodies against the macrophage marker F4/80 as well as GFP. This analysis showed that the hucMSCs were engulfed and phagocytosed by macrophages (Fig. 3B). We next investigated the effect of phagocytosing hucMSCs on macrophages. To this end, markers and cytokines related to macrophage phenotypes were assessed on days 7 and 14. *Nos2*, *Cxcl9*, and *Cxcl10* expression levels were dramatically elevated in mice that received hucMSCs compared to mice treated with bleomycin on day 7 (Fig. 3C–E). Meanwhile, *Mrc1*, *Arg1*, *Chil3*, and *Tgm2* expression levels were significantly decreased at day 14 following hucMSC injection (Fig. 3F–I). These results suggested that hucMSCs were phagocytosed by macrophages after their infusion which also led to a change in macrophage phenotype.

### Single cell RNA sequencing revealed a novel subtype of interferon-sensitive macrophages after hucMSC infusion

**Fig. 4:**
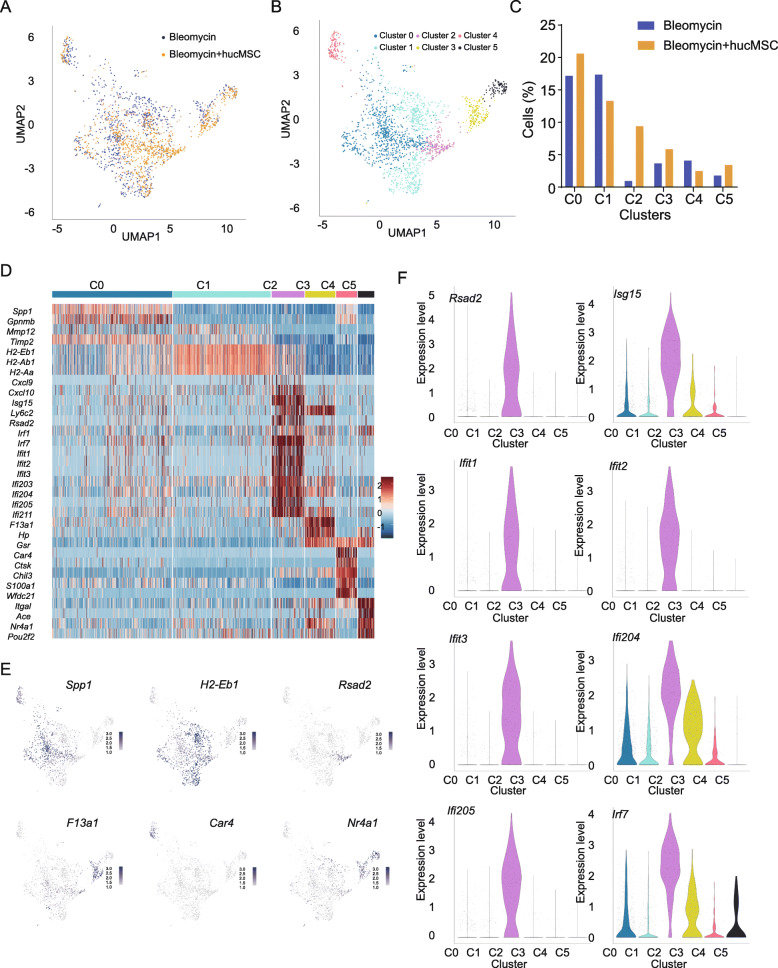
Single cell RNA sequencing revealed an interferon-sensitive macrophage population in hucMSCs infused lungs. **A** UMAP plot of macrophages from bleomycin- and hucMSC-treated mouse lungs. **B**, **C** The proportions of different macrophage clusters. **D** Heatmap of some differentially expressed genes between each macrophage cluster. **E** t-SNE visualization overlaid with the expression of *Spp1*, *H2-Eb1*, *Rsad2*, *F13a1*, *Car4*, and *Nr4a1*. **F** Violin plots of *Rsad2*, *Isg15*, *Ifit1*, *Ifit2*, *Ifit3*, *Ifi204*, *Ifi205*, and *Irf7* expression in each macrophage cluster

To gain mechanistic insight into the effects of hucMSCs on macrophages, we performed single cell RNA sequencing analysis to characterize the gene expression profiles of lung macrophages 7 days after treatment with either intratracheal bleomycin or hucMSC infusion, when lung regeneration is active. We analyzed profiles of 1845 total macrophages from all samples with sufficiently high gene expression signals after filtering, normalization, and removal of potential outliers (Fig. 4A). We identified six macrophage clusters through unbiased clustering in both treatments (Fig. 4B; Table S2) and all clusters expressed macrophage-specific markers, including *Adgre1*, *Lyz2*, and *CD68* (Figure S4A). All six detected clusters were detected at varying levels in both bleomycin-treated and hucMSC-treated mice: Cluster 0, cluster 1, cluster 3, and cluster 4 were present in bleomycin-treated and hucMSC-treated mice lungs at a relatively similar frequency. On the other hand, cluster 2 and cluster 5 were enriched in hucMSC-treated mice lung, especially for cluster 2 cells in which hucMSC-treated lungs have a 10-fold increase compared to bleomycin-treated lungs by day 7 (Fig. 4C). Cluster 0 was enriched for the expression of fibrosis-associated genes (*Spp1*, *Gpnmb*, *Mmp12*, and *Timp2*). Cluster 1 macrophages expressed high levels of antigen presentation-associated genes (*H2-Eb1*, *H2Ab1*, and *H2Aa*). Macrophages in cluster 3 highly expressed *F13a1*, *Hp*, and *Gsr*, which suggests that cluster 3 belongs to the monocyte subtype. Macrophages from cluster 4 expressed genes involved in the inflammatory response, cytokine production, and matrix metalloproteinase activation (*Car4*, *Ctsk*, *Chil3*, *S100a1*, and *Wfdc21*). Cluster 5 macrophages express high levels of the transcription factors *Nr4a1* and *Pou2f2*, which suggests they are the Ly6C^lo^ monocytes (Fig. 4D, E). Cluster 2 macrophages in particular expressed a strong type 1 IFN (interferon) signature, including the IFN-responsive genes (IRGs) *Rsad2*, *Isg15, Ifit1*, *Ifit2*, *Ifit3*, *Ifi204*, *Ifi205*, and *Irf7* (Fig. 4F), and other IRGs were also highly expressed in IFNSMs (herein referred to as “IFN-sensitive macrophages”). Based on the ontology analysis of marker gene expression, IFNSMs were different from other macrophage clusters. Thus, our analysis demonstrated the emergence of a unique IFNSM cluster in mouse lungs following hucMSC infusion.

### hucMSCs promote interferon-responsive gene expression in macrophages

**Fig. 5:**
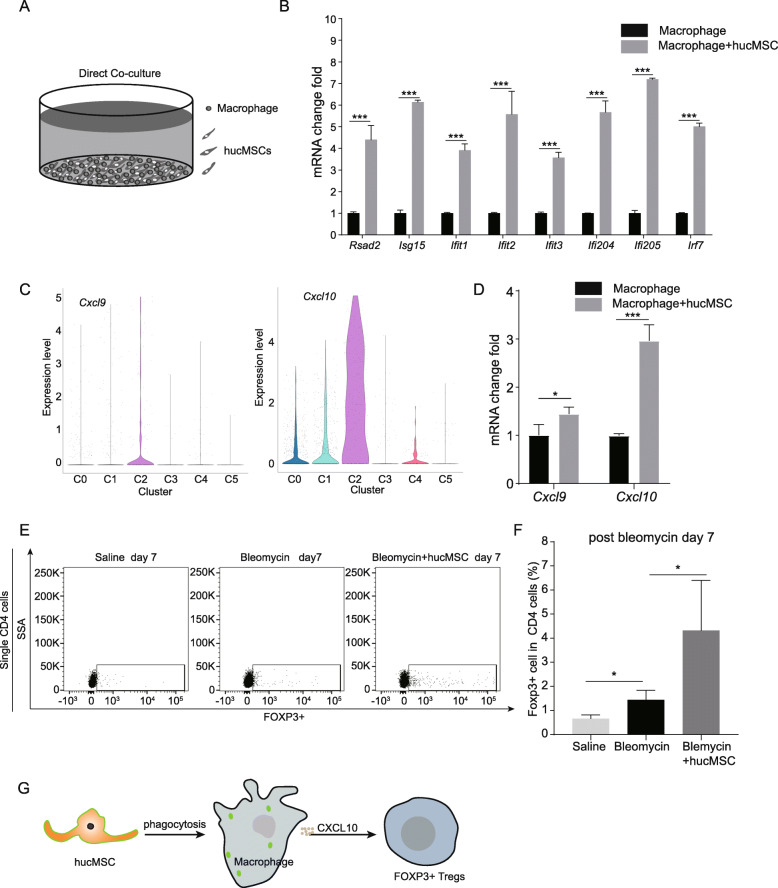
Macrophage and hucMSCs interaction increased interferon-responsive gene expression in macrophages. **A** Diagram of macrophage and hucMSCs co-culture. **B** *Rsad2*, *Isg15*, *Ifit1*, *Ifit2*, *Ifit3*, *Ifi204*, *Ifi205*, and *Irf7* expression levels in macrophage co-cultured hucMSCs (mean ± SD, *n* = 3 wells per group). **C** Violin plots of *Cxcl9* and *Cxcl10* in each macrophage cluster. **D** *Cxcl9* and *Cxcl10* expression levels in macrophages co-cultured with hucMSCs (mean ± SD, *n* = 3 wells per group). **E** Flow cytometry analysis of Foxp3 regulatory T cells in saline-treated mice lungs, bleomycin-treated mice lungs, and hucMSC-treated mice lung. **F** Quantification of Foxp3-positive Tregs in saline-treated mice lungs, bleomycin-treated mice lungs, and hucMSC-treated mice lungs at day 7 (mean ± SD, *n* = 3 mice per group). **G** Working model showing that hucMSCs interact with macrophage to recruit Tregs into lung. *p < 0.05, ***p < 0.001, n.s. no significant difference, Student’s *t*-test

Our observation of the emergence of IFNSMs after hucMSC infusion encouraged us to examine the relationship between hucMSC infusion and IFNRMs. To better understand how hucMSCs regulate macrophage behavior, we isolated CD45+; F4/80+ macrophages from mice treated with bleomycin after 1 day by FACS and co-cultured these cells with hucMSCs (Fig. 5A). After 3 days of co-culture, the expression level of interferon-responsive genes significantly increased in macrophages co-cultured with hucMSCs (Fig. 5B). This suggested that the direct interaction between hucMSCs and macrophages elevated interferon-responsive gene expression in macrophages in vitro co-culture. To investigate the function of IFNSMs, we analyzed the characteristic genes according to their expression level and subcellular locations. We found that *Cxcl10* was the most highly expressed secretory protein in the IFNSMs (Fig. 5C). Meanwhile, *Cxcl9* and *Cxcl10* were also increased in the macrophages after co-culturing with hucMSCs (Fig. 5D). CXCL10 is involved in cell proliferation, migration, and angiogenesis [17]. The receptor of CXCL10 is CXCR3, which is highly expressed in the regulatory T cells (Treg) [11, 22]. To verify whether there was an increased number of Tregs in mice after hucMSC infusion, we compared the numbers of Tregs in mice treated with bleomycin and hucMSCs on day 7. Flow cytometry indicated that there were more FOXP3 Tregs in hucMSCs injected mice lung (Fig. 5E, F). These data suggest that hucMSCs induced the increase of *Cxc10* expression in macrophages and enhance the recruitment of Tregs to the lungs (Fig. 5G).

## Discussion

In this study, we found that hucMSC infusion could ameliorate bleomycin-induced PF. Though hucMSCs disappeared rapidly in the lungs following intravenous administration, hucMSCs still exerted therapeutic effects such as decreased collagen deposition and mortality. hucMSCs were phagocytosed by macrophages and altered their phenotype in the lungs and a unique macrophage cluster that sensitive to interferons was identified by single cell RNA sequencing. This might be due to the phagocytosis of MSCs changing the sensitivity of macrophages to interferons. The phagocytosis of MSCs by macrophages is consistent with previous reports. The phagocytosis of MSCs changed the transcriptional profile of macrophages and, surprisingly, propagated the function of MSCs despite MSCs no longer being present in vivo. In the graft versus host disease model, macrophages engulf apoptotic MSCs and produce indoleamine 2,3-dioxygenase, which then performs its immunosuppressive function [9]. MSCs could also transfer mitochondrial and microRNA into the macrophage and enhance the bioenergetics of macrophage [18, 29]. Macrophages can also engulf cytoplasmic components of MSCs which might downregulate genes involved in antigen presentation and suppress the activation of helper T cells [6]. MSC-derived exosomes could change the proportion of infiltrating classical and nonclassical monocytes in the lungs after bleomycin treatment and revert the PF [25, 29].

CXCL9 and CXCL10 are chemokines of the CXC subfamily, whose main function is promoting the trafficking of various leukocytes and mobilizing lung mesenchymal progenitor cells, regulating angiogenesis, and vascular remodeling [35]. CXCL10-deficient mice have dramatically increased fibroblast accumulation and more severe PF [15, 36]. The deficiency of the CXCL9 and CXCL10 receptor, CXCR3, also causes a similar phenotype [14]. Systemic administration of CXCL10 can reduce PF and inhibit the deposition of extracellular matrix by regulating angiogenesis [15]. Elevated *Cxcl10* expression in macrophages following hucMSC infusion recruits Tregs into the lungs which may partially explain how hucMSCs perform their immunosuppressive function. *CXCR3* was also expressed in some alveolar type 2 cells [12], whose function remains to be investigated.

hucMSC infusion does not affect the number of macrophages day 7 after bleomycin treatment (Figure S5A-5B). However, there are decreased macrophage numbers at day 14 after bleomycin treatment, and both alveolar macrophages and interstitial macrophages are decreased at day14 (Figure S5C-5F). Fewer macrophages lead to the decreased expression level of *Mrc1* (encode CD206) and *Chil3* (Fig. 3F, H), which play profibrotic roles during the repair phase. Administration of CD206 blocking peptide inhibits bleomycin-induced PF, which suggests that CD206 may be an interesting target for the treatment of fibrosis [10]. Chitinase 3-like 1 (coding by *Chil3*) is a prototypic chitinase-like protein that was elevated in patients with idiopathic PF [21, 42]. In the bleomycin-induced lung fibrosis model, *Chil3* expression was transiently decreased at the acute injury phase and then elevated at the fibrotic phase [42]. hucMSC infusion decreased macrophages at the fibrotic phase, which may also contribute to the alleviation of fibrosis caused by bleomycin.

PF is an important public health problem that leads to high mortality rates and an economic burden on patients. Here, we found that hucMSCs attenuated fibrosis induced by bleomycin and identified a subtype of interferon-sensitive macrophages. Our study suggests that hucMSCs perform immunosuppressive functions by recruiting regulatory T cells, partially through their interaction with macrophages. Future investigations that combine single cell RNA sequencing and in vivo tracing methods will clarify the mechanism of MSC in clinical models.

## Conclusion

In the current study, we found hucMSCs can attenuate pulmonary fibrosis via macrophage. After hucMSC infusion, a subset of macrophage exhibited increased expression of interferon-responsive genes was identified by single cell RNA sequencing. This subtype of macrophages had increased *Cxcl10* expression that recruited more Tregs into the lung, which partially explains how the hucMSCs perform their immunosuppressive function.

## Supplementary Information

**Additional file 1: Figure S1. Characterization of phenotype and differentiation potential of hucMSCs.** A. Morphology of cultured hucMSCs. (B-I) Surface antigen expression on hucMSCs by flow cytometry. Cells were stained with antibodies against mouse IgG1 isotype FITC (B), mouse IgG1 isotype PE (C), mouse IgG1 isotype APC (D), CD90 FITC (E), CD73 PE (F), CD105 APC (G), CD45 FITC (H), CD34 PE (I). (J-L) Differentiation potential of hucMSCs into adipocytes (J), chondrocytes (K), and osteocytes (L). **Figure S2. Gating strategy of lung fibroblast**. (A) Representative gating strategy of EpCAM-CD31-CD45- lung fibroblast. **Figure S3.** ***In vivo*** **imaging of DiR-labeled hucMSCs distribution**. (A) Control mice (saline-treated) and bleomycin-treated mice were injected with DiR-labeled hucMSCs at day 0, and imaged at day 1, day 3, day 5, day 7, and day 14. **Figure S4. Macrophage markers expression on a t-SNE plot**. (A) Normalized expression of macrophage markers *Adgre1*, *Cd68*, and *Lyz2* overlaid on a t-SNE plot. **Figure S5. Dynamics of lung macrophage after bleomycin treatment**. (A) Representative gating strategy of macrophages. (B) Quantification of lung macrophages in saline-treated mice lungs, bleomycin-treated mice lungs, and hucMSCs-treated mice lungs at day 7 and day 14. (C) Representative gating strategy of alveolar macrophage. (D) Quantification of alveolar macrophages in saline-treated mice lungs, bleomycin-treated mice lungs, and hucMSCs-treated mice lungs at day 7 and day 14 (mean ± SD, *n* = 3 mice per group). (E) Representative gating strategy of interstitial macrophages and monocytes. (F) Quantification of interstitial macrophages and monocytes in saline-treated mice lungs, bleomycin-treated mice lungs, and hucMSCs-treated mice lungs at day 7 and day 14 (mean ± SD, *n* = 3 mice per group). *p < 0.05, ***p < 0.001, n.s. no significant difference, Student’s *t*-test.

**Additional file 2:** Cell type annotation and differentially expressed genes in each cluster.

**Additional file 3:** List of significantly upregulated gene in each cluster of hucMSCs-treated mice compared with bleomycin-treated mice.

## Data Availability

The datasets used and/or analyzed during the current study are available from the corresponding author on reasonable request.

## Abbreviations

hucMSC: Human umbilical mesenchymal stromal cell
PF: Pulmonary fibrosis
ECM: Extracellular matrix
AM: Alveolar macrophage
IM: Interstitial macrophage
Treg: Regulatory T cell
mGFP: Membrane green fluorescent protein
IFNSM: IFN-sensitive macrophage
