# Supplementary figures and images for: Human umbilical cord mesenchymal stromal cells attenuate pulmonary fibrosis via regulatory T cell through interaction with macrophage
Source: Stem Cell Res Ther. 2021 Jul 13;12:397. doi: 10.1186/s13287-021-02469-5 (PMC8278716; doi:10.1186/s13287-021-02469-5)

Figure 1

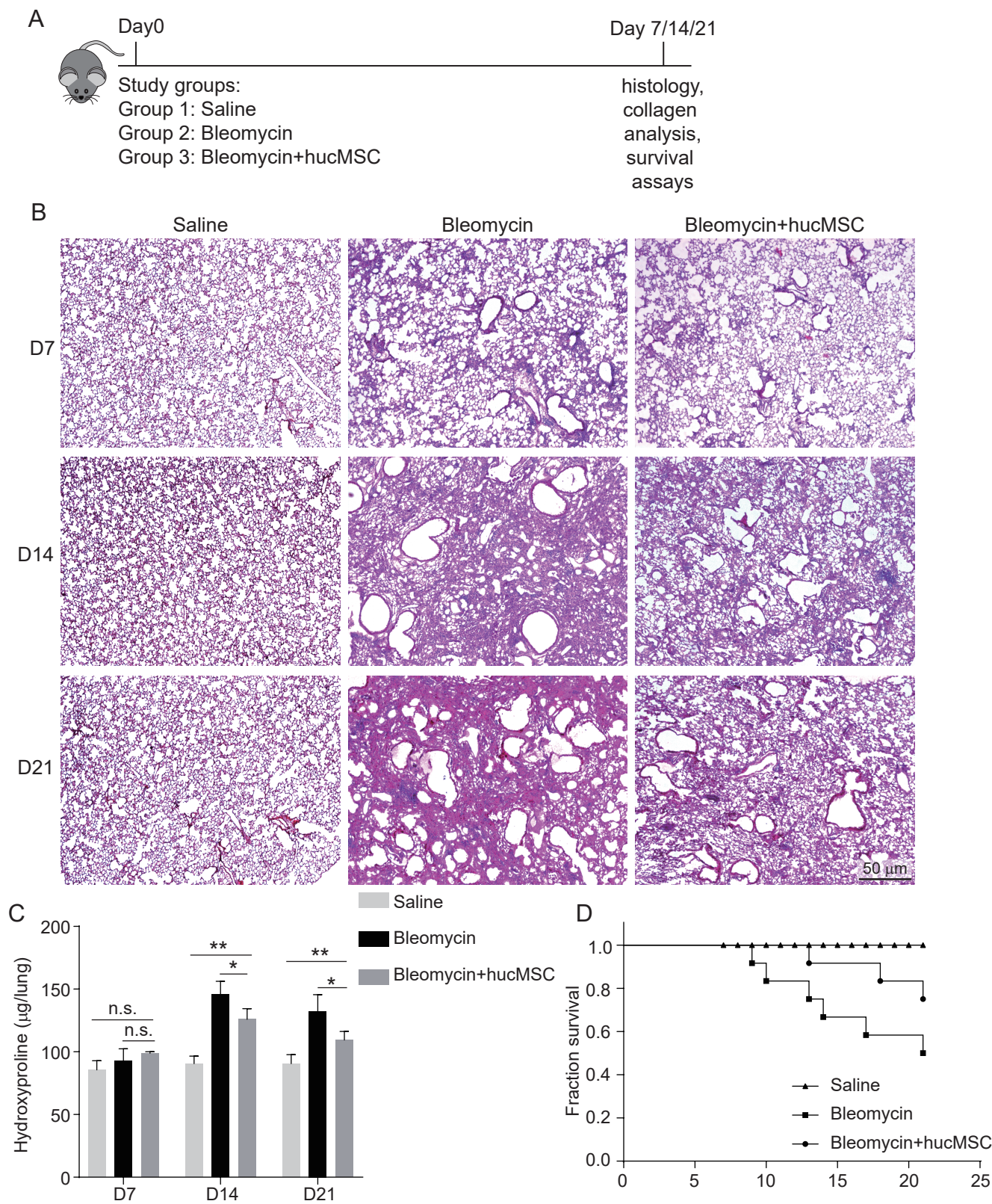

Figure 2

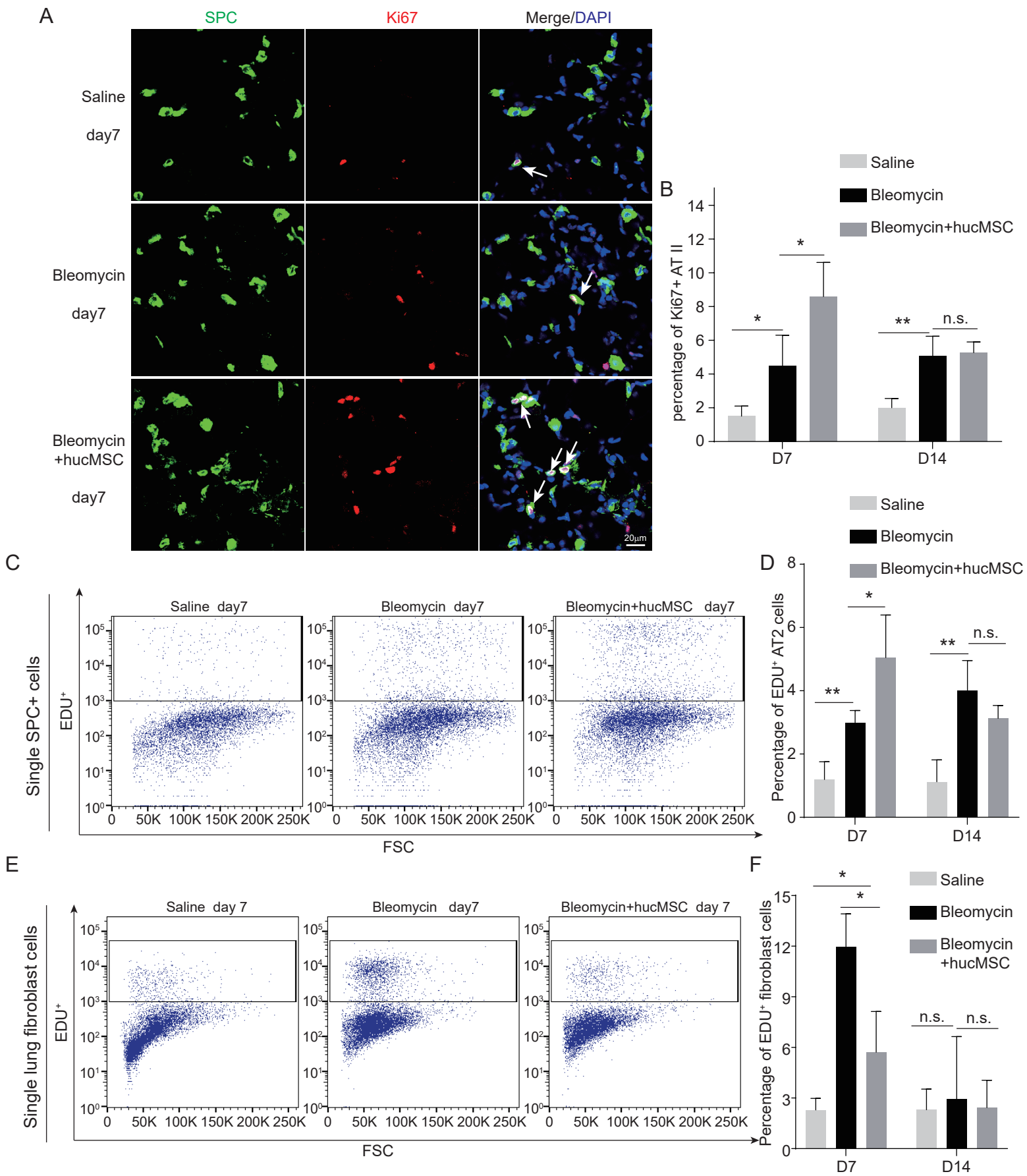

Figure 3

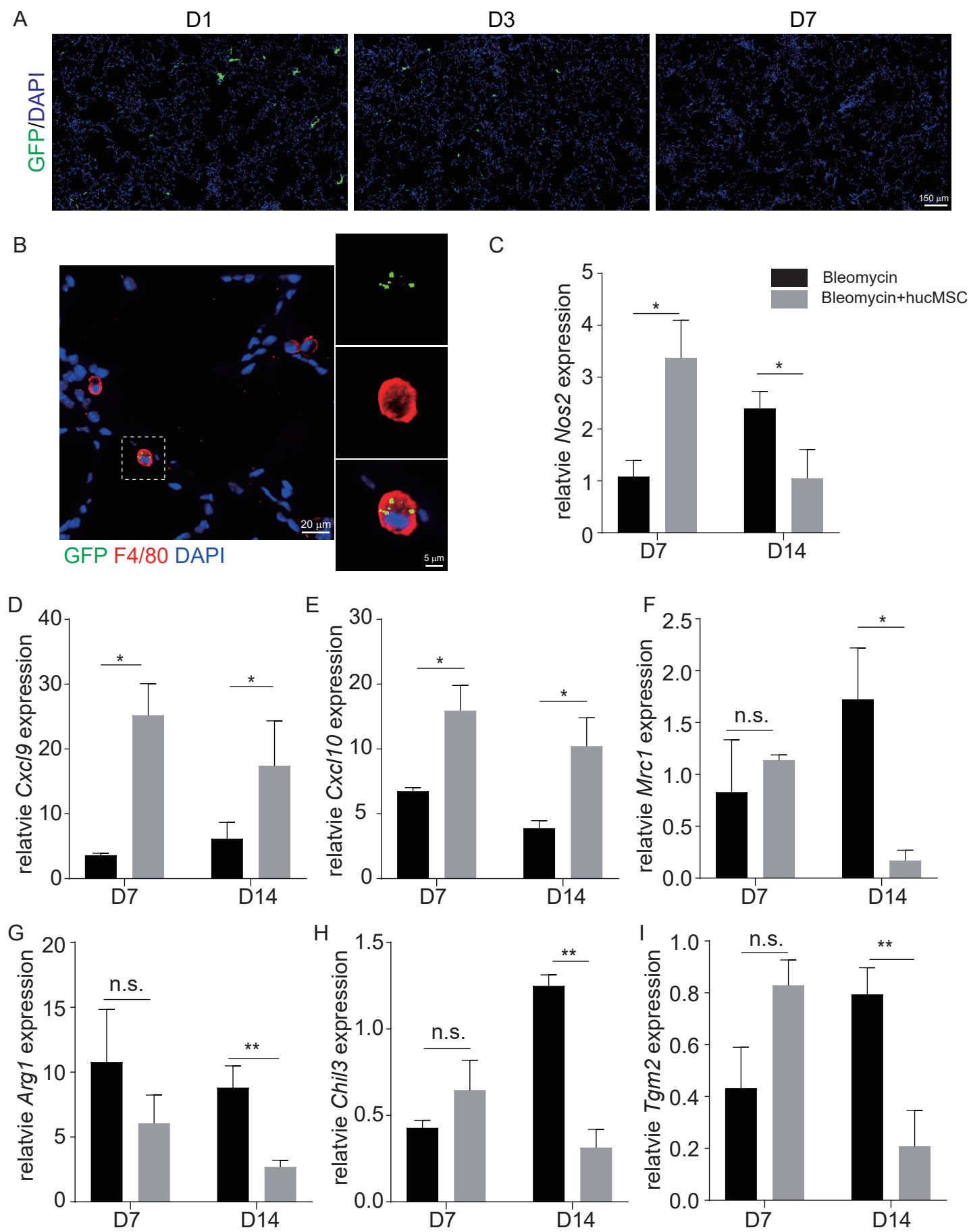

Figure 4

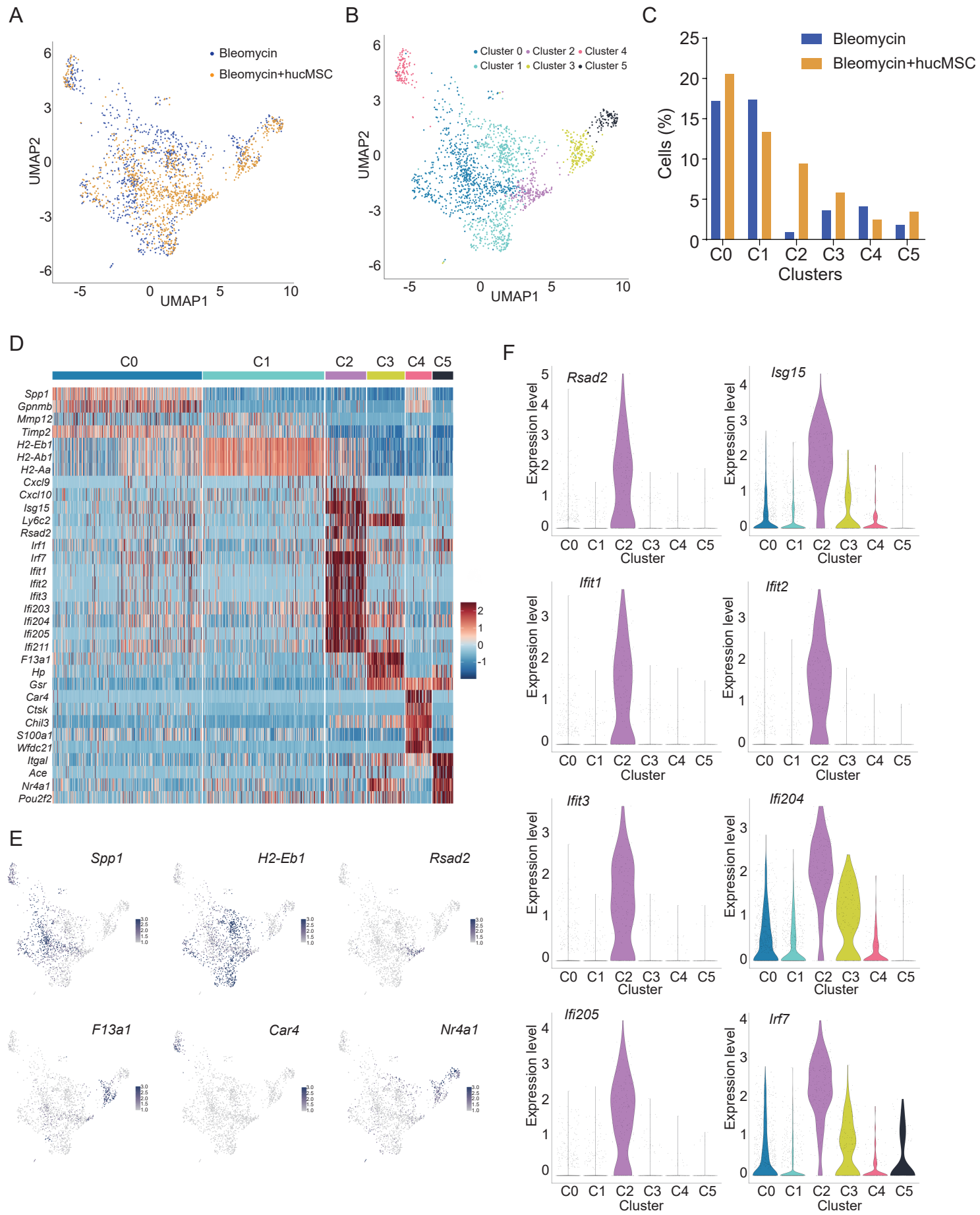

Figure 5

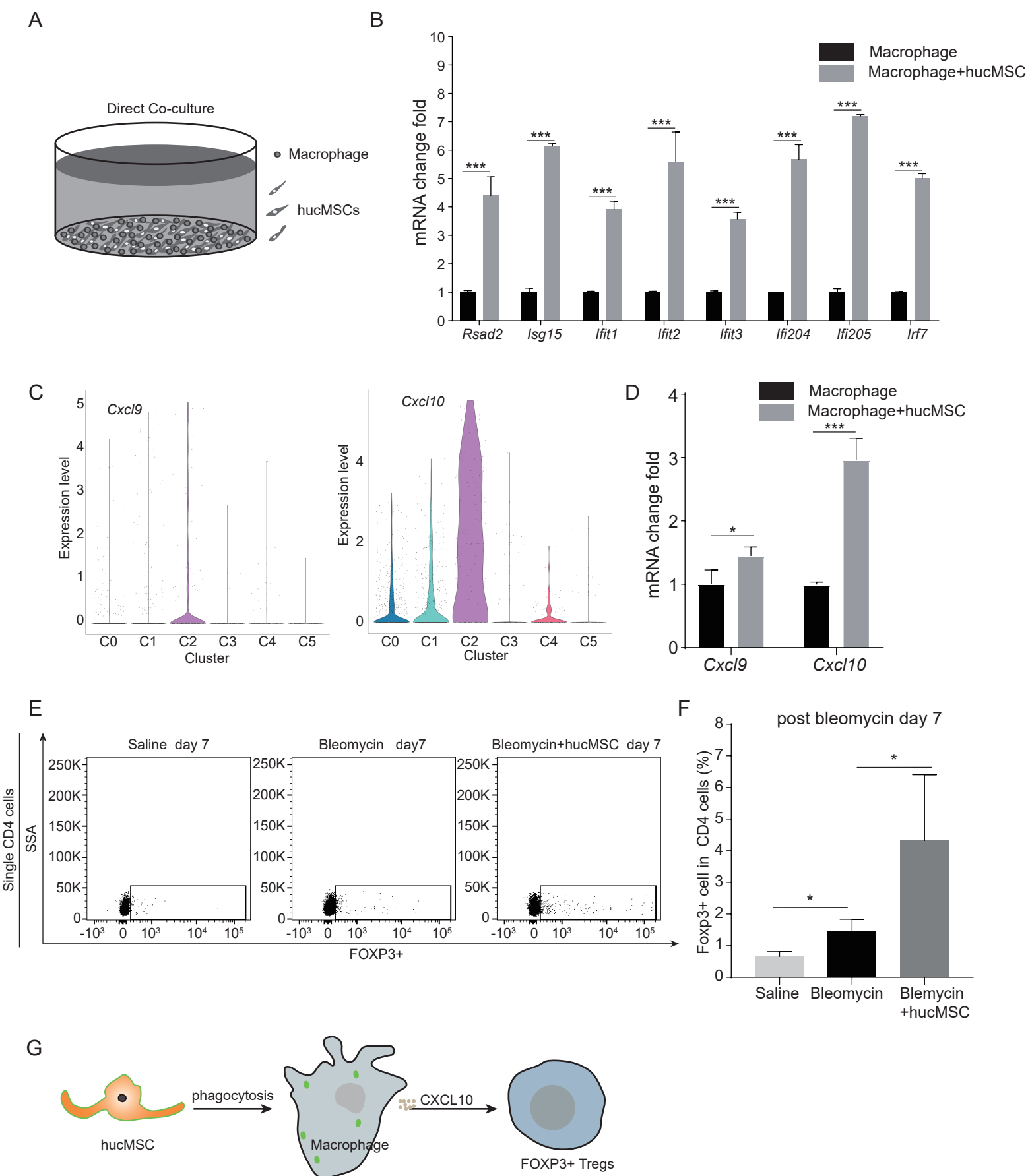

Figure S1

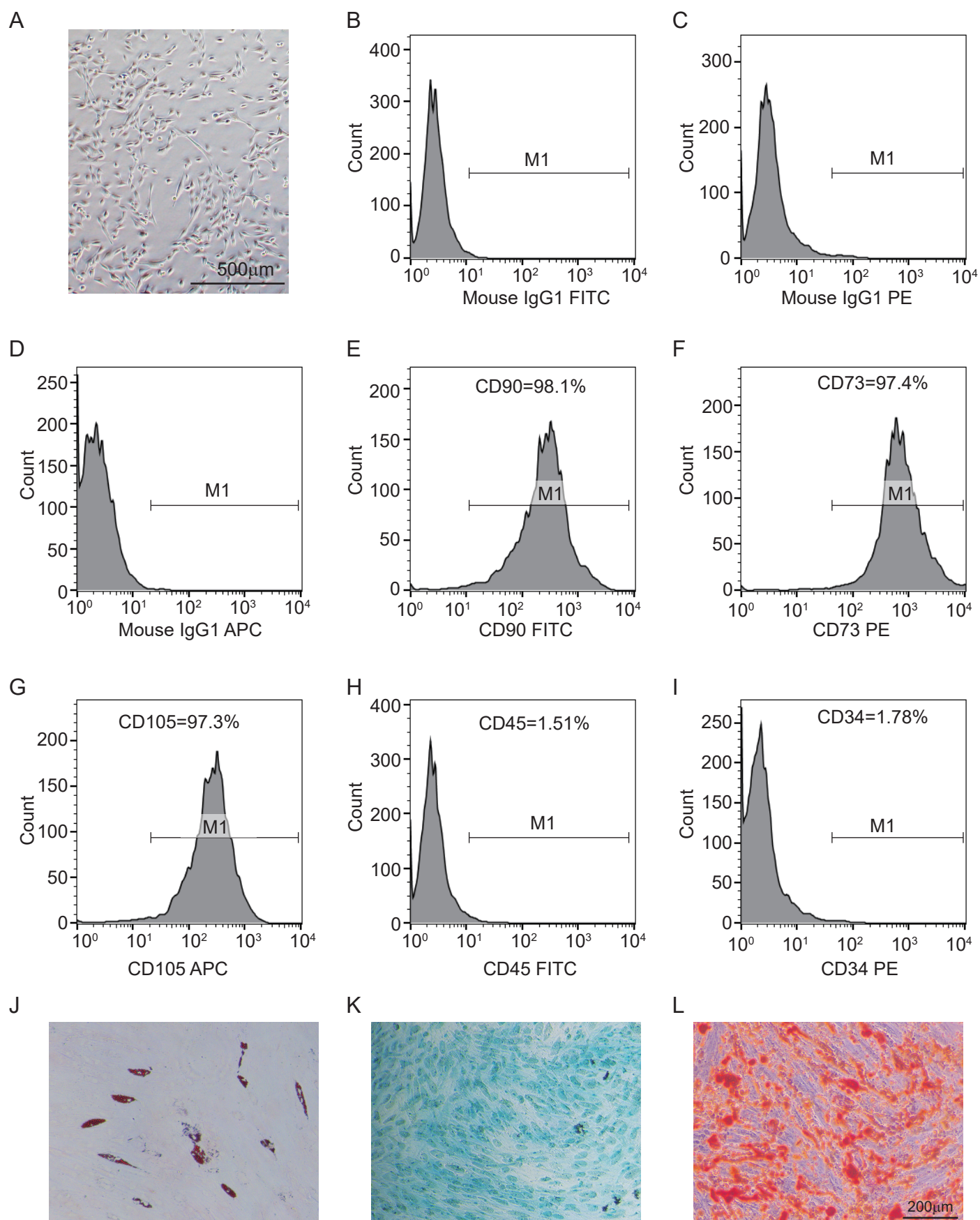

Figure S2

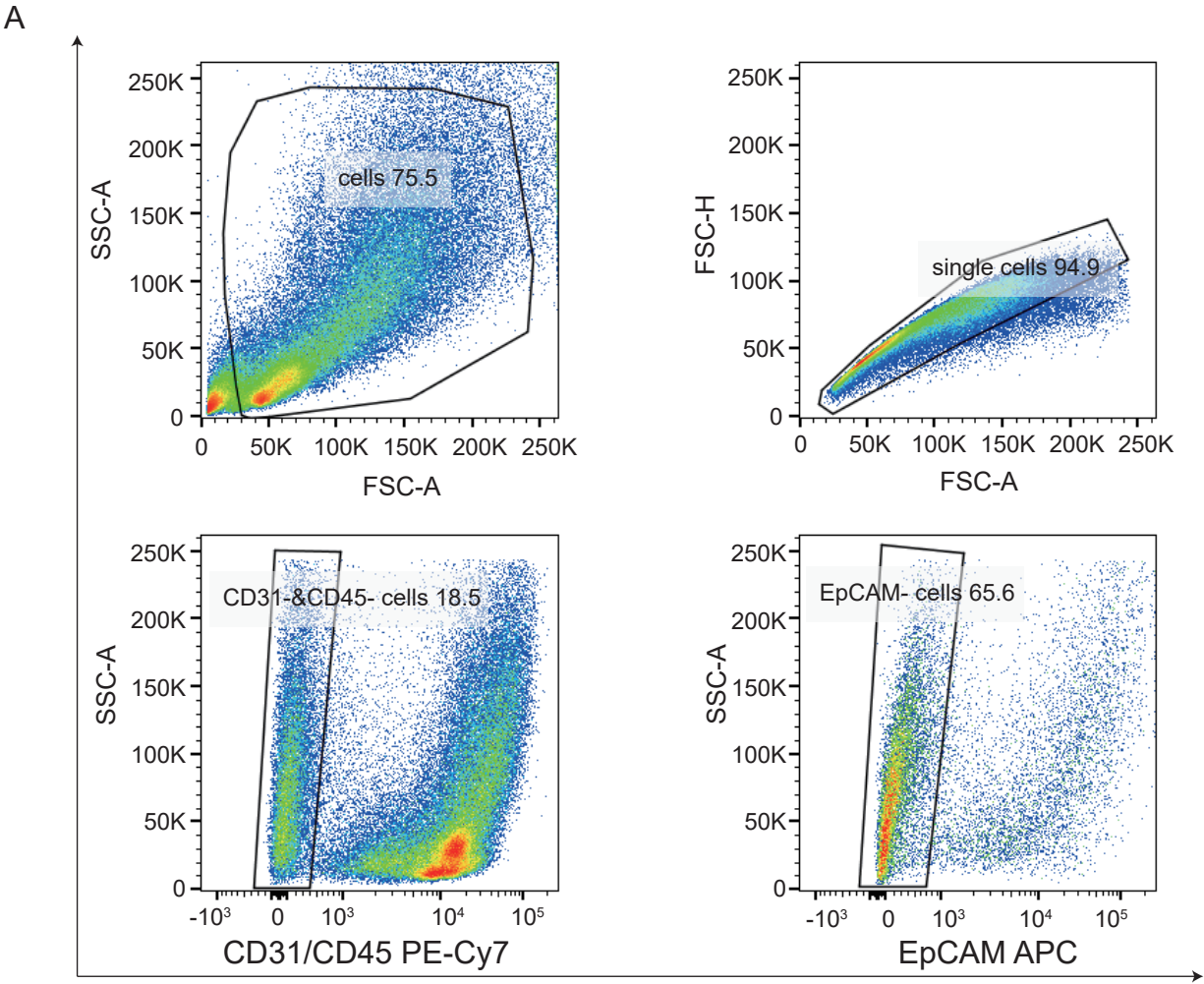

Figure S3

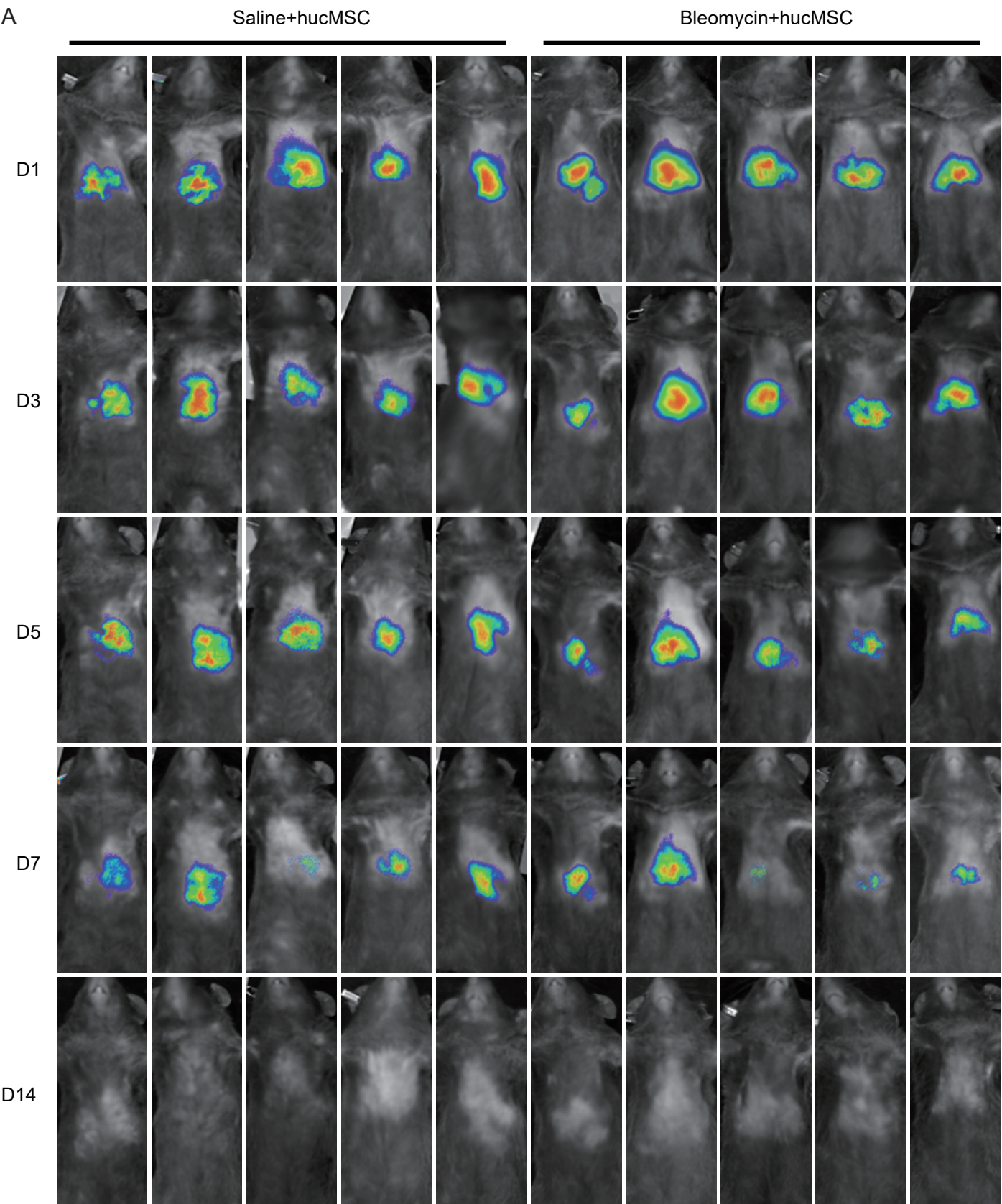

Figure S4

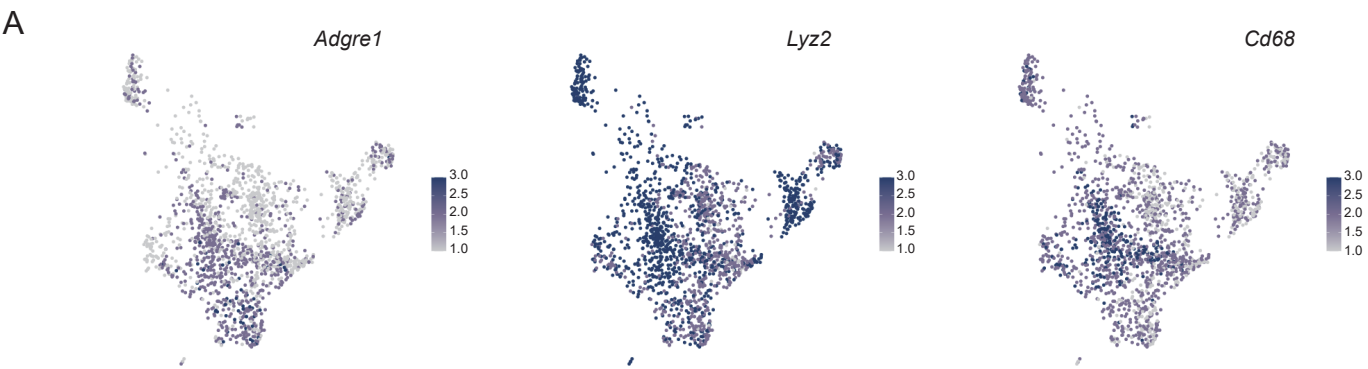

Figure S5

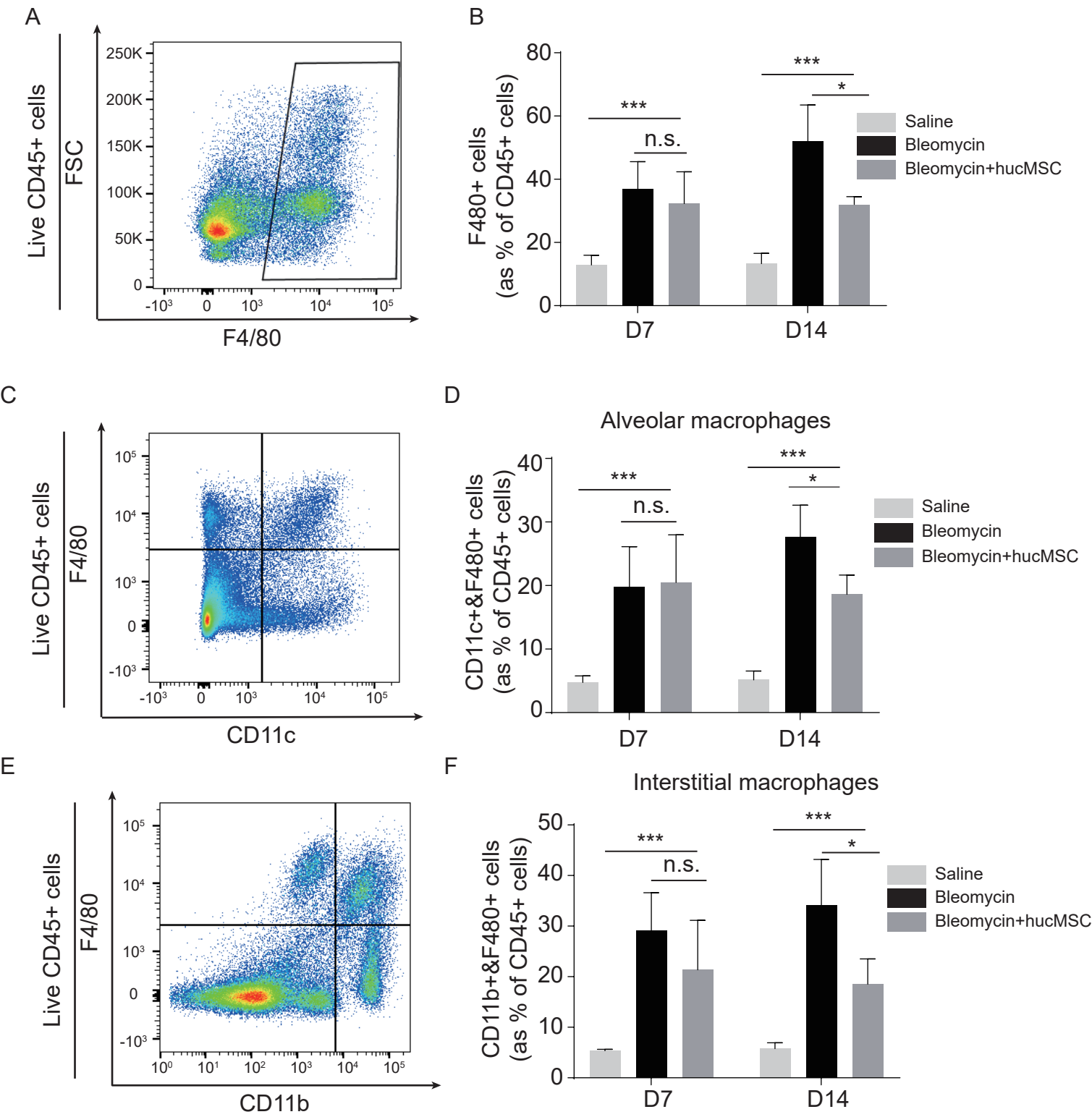

Supplement: Supplementary file 1 — Additional file 1: Figure S1. Characterization of phenotype and differentiation potential of hucMSCs. A. Morphology of cultured hucMSCs. (B-I) Surface antigen expression on hucMSCs by flow cytometry. Cells were stained with antibodies against mouse IgG1 isotype FITC (B), mouse IgG1 isotype PE (C), mouse IgG1 isotype APC (D), CD90 FITC (E), CD73 PE (F), CD105 APC (G), CD45 FITC (H), CD34 PE (I). (J-L) Differentiation potential of hucMSCs into adipocytes (J), chondrocytes (K), and osteocytes (L). Figure S2. Gating strategy of lung fibroblast. (A) Representative gating strategy of EpCAM-CD31-CD45- lung fibroblast. Figure S3. In vivo imaging of DiR-labeled hucMSCs distribution. (A) Control mice (saline-treated) and bleomycin-treated mice were injected with DiR-labeled hucMSCs at day 0, and imaged at day 1, day 3, day 5, day 7, and day 14. Figure S4. Macrophage markers expression on a t-SNE plot. (A) Normalized expression of macrophage markers Adgre1, Cd68, and Lyz2 overlaid on a t-SNE plot. Figure S5. Dynamics of lung macrophage after bleomycin treatment. (A) Representative gating strategy of macrophages. (B) Quantification of lung macrophages in saline-treated mice lungs, bleomycin-treated mice lungs, and hucMSCs-treated mice lungs at day 7 and day 14. (C) Representative gating strategy of alveolar macrophage. (D) Quantification of alveolar macrophages in saline-treated mice lungs, bleomycin-treated mice lungs, and hucMSCs-treated mice lungs at day 7 and day 14 (mean ± SD, n = 3 mice per group). (E) Representative gating strategy of interstitial macrophages and monocytes. (F) Quantification of interstitial macrophages and monocytes in saline-treated mice lungs, bleomycin-treated mice lungs, and hucMSCs-treated mice lungs at day 7 and day 14 (mean ± SD, n = 3 mice per group). *p < 0.05, ***p < 0.001, n.s. no significant difference, Student’s t-test. [file 13287_2021_2469_MOESM1_ESM.pdf]
